# The Invisible Threat of Antibiotic Resistance in Food

**DOI:** 10.3390/antibiotics14030250

**Published:** 2025-03-01

**Authors:** Gabriella Kiskó, Belma Bajramović, Fatma Elzhraa, Patrícia Erdei-Tombor, Viktória Dobó, Csilla Mohácsi-Farkas, Andrea Taczman-Brückner, Ágnes Belák

**Affiliations:** 1 Department of Food Microbiology, Hygiene and Safety, Institute of Food Science and Technology, Hungarian University of Agriculture and Life Sciences, H-1118 Budapest, Hungary; kisko.gabriella@uni-mate.hu (G.K.); belma.bajramovich@gmail.com (B.B.); dr.fatmaelzhraa@mans.edu.eg (F.E.); tombor.patricia@phd.uni-mate.hu (P.E.-T.); dobo.viktoria01@gmail.com (V.D.); mohacsine.farkas.csilla@uni-mate.hu (C.M.-F.); belak.agnes@uni-mate.hu (Á.B.); 2 Department of Food Hygiene and Control, Faculty of Veterinary Medicine, Mansoura University, Mansoura 35516, Egypt

**Keywords:** antibiotics, resistance, foods, bacteria, gene transfer, potential risk

## Abstract

The continued and improper use of antibiotics has resulted in the emergence of antibiotic resistance (AR). The dissemination of antibiotic-resistant microorganisms occurs via a multitude of pathways, including the food supply. The failure to comply with the regulatory withdrawal period associated with the treatment of domestic animals or the illicit use of antibiotics as growth promoters has contributed to the proliferation of antibiotic-resistant bacteria in meat and dairy products. It was demonstrated that not only do animal and human pathogens act as donors of antibiotic resistance genes, but also that lactic acid bacteria can serve as reservoirs of genes encoding for antibiotic resistance. Consequently, the consumption of fermented foods also presents a potential conduit for the dissemination of AR. This review provides an overview of the potential for the transmission of antibiotic resistance in a range of traditional and novel foods. The literature data reveal that foodborne microbes can be a significant factor in the dissemination of antibiotic resistance.

## 1. Introduction

Resistance of microorganisms to antimicrobial agents can either be natural or acquired. Natural resistance is a stable, heritable trait specific to species or larger taxa. Acquired resistance is a change in the natural susceptibility spectrum within a generation that can be acquired through chromosome mutation, but the most common mechanisms rely on mobile genetic elements (MGEs) such as plasmids, transposons, and integrons [1]. These MGEs can be horizontally transferred between different genera, even between pathogenic species, through conjugation, transformation, or transduction. The bacterial resistance can happen via alteration of the target sites of drugs, decreasing membrane permeability, active efflux of drugs, external factors, and inactivating or modifying the antimicrobial agent [2].

Antimicrobials are compounds that are used to kill or stop the growth of harmful microorganisms and prevent or treat infections. As a consequence of the usage, overuse, and misuse of antimicrobials, antimicrobial resistance has developed. AMR occurs when microorganisms are exposed to an agent that inhibits their growth for a prolonged period of time or at very low concentrations and as a result, they are altered. The change leads to more resistant microorganisms to the particular agent so that the agent used against these microbes is no longer effective. A priority area of antimicrobial resistance (AMR) is antibiotic resistance (AR). Antibiotics are antimicrobial substances that have the capacity to inhibit the growth of microorganisms or kill them, and are widely used for the treatment of bacterial infections in humans and animals, as well as in non-medical applications [3]. Antibiotic resistance is used to define the innate ability of microorganisms to multiply in the presence of high concentrations of an antibiotic, regardless of the time of exposure, and is expressed as the Minimum Inhibitory Concentration (MIC) [4].

The golden age of efficient use of antibiotics in human medicine dates back to the 1940s–1980s. The non-therapeutic use of antibiotics in food-producing animals as growth promoters accelerated the spread of antibiotic-resistant bacteria [5], resulting in a threat to human health throughout the food chain [6,7]. In recent decades, the consumption of antibiotics has increased massively, partly because application of antibiotics in veterinarian practice has expanded [8].

The continuous and indiscriminate use of antibiotics has resulted in the emergence of antibiotic-resistant bacteria, which has contributed to a significant increase in mortality from multidrug-resistant bacterial infections. This has led to a major public health crisis on a global scale [9,10].

A recent study [11] provides an estimation for the global trends in the use of antibiotics in food animals between 2017 and 2030. They estimated that sales are expected to increase by 11.5% by 2030. However, Harbarth et al. [12] provide a projection of antibiotic use for livestock in India, where the use of quinolones is expected to increase up to 243% by 2030.

According to a collaborative report by the European Centre for Disease Prevention and Control (ECDC), the European Food Safety Authority (EFSA) and the European Medicines Agency (EMA) [13] the overall usage of antibiotics between 2016 and 2018 was lower for the first time in food-producing animals compared to human applications in Europe as a result of state-level measures to reduce antibiotics in food-producing animals.

To reduce the emergence of antibiotic-resistant microorganisms the World Health Organization (WHO) has recommended the “complete restriction of the use of antibiotics in animals to promote growth or prevent disease in the absence of diagnosis”, and the Food and Drug Administration [14] has approved antibiotics in food animals only for the treatment, control, and prevention of disease. Recently, China, the world’s largest consumer of antibiotics in livestock animals, adopted a national plan to reduce the use of antibiotics in animal feed [15].

Both animal and human pathogens serve as donors of antibiotic resistance genes (ARG) to pathogens that infect humans [12]. Antibiotic-resistant bacteria (ARB) can spread from food as well. For example, the use of fluoroquinolones (e.g., enrofloxacin) in food-producing animals resulted in the spread of ciprofloxacin-resistant *Salmonella*, *Campylobacter* and *E. coli* [10]. The problem can be intensified if microorganisms become resistant to several antibiotics at the same time, i.e., the development of multidrug resistance (MDR). “*MDR is defined as non-susceptibility to at least one agent in three or more antimicrobial categories and up to (and including) the total number of all antimicrobial categories minus two*” [16].

Epidemiological studies report a significant acceleration in the evolution and spread of multidrug-resistant bacteria (MRB) over the past 50 years. The inappropriate use of antibiotics and the ability of bacteria to transmit resistance determinants have amplified the problem [17].

It is evident that both foods produced in accordance with traditional methods and those classified as new foods are significant sources of nutrients in human nutrition. Nevertheless, in addition to their physiological importance for humans, the consumption of these foods can also pose a potential hazard. It is imperative to acknowledge the inherent presence of microorganisms in food, particularly in the context of fermented products. However, microbes not only interact with their environment, but also with members of other populations. Through genetic information transfer pathways, microbes can equip themselves with newer and newer properties that help them survive in changing environments. Such a property can also be resistance to antibiotics, which can be demonstrated with increasing frequency, for example, in the case of lactic acid bacteria. The purpose of this article was to review the potential risks to consumers from the consumption of traditional and novel foods, primarily due to the ever-increasing spread of antibiotic-resistant bacteria.

## 2. Antibiotics and Their Effects on Bacterial Resistance

In recent decades, a number of mechanisms related to the behavior of bacteria in relation to antimicrobial compounds have been observed and described. It is essential to define these mechanisms in order to gain a precise understanding and to facilitate examination of bacterial responses. The mechanisms in question include resistance, tolerance, and persistence.

At present, the published definitions of antibiotic resistance vary considerably. The term “resistance” is employed to denote various characteristics exhibited by bacteria, which can be categorized in accordance with their origin (intrinsic versus acquired resistance) or type (single, multiple, or cross-resistance). These characteristics encompass phenotypic traits, such as growth patterns, and genotypical traits, including the presence and/or expression of specific genes [18].

The term “tolerance” is used to denote the ability of microorganisms to survive transient exposure to high concentrations of an antibiotic without a change in MIC. This process is often achieved by slowing down an essential bacterial process. Tolerance confers upon bacterial cells the capacity to withstand transient exposure to antibiotic concentrations that would otherwise prove lethal [4].

The term “persistence” is employed to denote the ability of a subpopulation of a clonal bacterial population to survive exposure to high concentrations of an antibiotic [4].

As a consequence of antibiotic resistance, more than 2.8 million infections caused by antibiotic-resistant bacteria occur in the U.S. yearly, resulting in more than 35,000 deaths, and it was predicted that the number of deaths worldwide will reach up to 10 million per year by 2050 [19]. Antibiotics are becoming less effective due to the increasing number of pathogenic strains with MDR. A decline in the effectiveness of antibiotics used to treat infectious diseases has led to an increase in mortality rates and prolonged hospital stays [20]. Resistance to antibiotics has led to at least one million deaths each year since 1990, with increasing rates of drug-resistant infections expected to claim more than 39 million lives between now and 2050 without further policy action, according to a landmark study by the Global Research on Antimicrobial Resistance (GRAM) Project [21]. The emergence of ARB is a major issue around the world, particularly within Europe [22]. The continuous emergence of antibiotic resistance additionally has increased the financial burden on healthcare systems [23,24].

The main determinants of antibiotic resistance are ARGs. Available data provide growing evidence that ARGs are widely distributed in different environmental patterns. For example, one of the resistance-causing *mcr1* genes was discovered in late 2015 [25]. Soon after its discovery, it was detected in 57 countries on 5 continents in almost all food-producing animals [19,26]. Health institutions have expressed concerns about the transmission of extended-spectrum β-lactamase (ESBL)-producing *E. coli* isolates, particularly in meat products, from the food chain to humans [22,27]. A Dutch study reported the transfer of ARGs from poultry to the human gut microbiota [28].

Antibiotic residues can cause various adverse health effects. Many antibiotics can cause allergic reactions, including anaphylactic shock. Sulfamethazine, oxytetracycline, and furazolidone can result in carcinogenicity; gentamicin may produce mutagenicity and nephropathy; chloramphenicol may lead to hepatotoxicity, reproductive disorders, and bone marrow toxicity [29,30]. Furthermore, therapeutic doses of antibiotics temporarily change both the composition of the human gastrointestinal microbiota and the immune and metabolic health of the host [31].

In addition to these serious consequences, low doses of antibiotics in feed or food and sub-lethal or sub-therapeutic doses also contribute to resistance and the emergence of ARB by promoting genetic and phenotypic variability in exposed bacteria [32]. It was demonstrated that the number of *Staphylococcus* and *Enterobacteriaceae* resistant to streptomycin, methicillin, tetracycline, and gentamicin is high in meat, meat products, and the milk of cows treated with sub-therapeutic concentrations of antibiotics in South Africa [33]. Several other studies also confirm the link between low antibiotic exposure and the development of resistance. These include many milk-related publications. In a Greek study, tetracycline resistance was 50% in *E. coli* strains isolated from cheese [34]. A Chinese study reported the resistance of *Bacillus cereus* strains isolated from milk to ampicillin (99%), penicillin (99%), and cefoxitin (95%) [35]. In an assay of Iranian milk samples, pathogenic *E. coli* isolates showed high resistance to tetracyclin (84%) and penicillin (46%) [36]. Likewise, high levels of resistance of pathogenic isolates were found in milk samples from other areas of Iran [37]. *Salmonella* isolates were highly resistant to penicillin (100%), cephalexin (100%), and amoxicillin (71.42%), while isolates of *S. aureus* were highly resistant to amoxicillin (100%), cephalexin (100%), and penicillin (84.00%). Moreover, 67% of pasteurized and unpasteurized milk samples from Kenya [38] contained ampicillin- and/or tetracycline-resistant *E. coli*. In the case of milk samples examined in Indonesia [39], the prevalence of *S. aureus* was 55.2%, and that of *E. coli* was 70.4%. A total of 7.4% of the isolates contained the *mecA* gene (MRSA) and 100% of the *E. coli* strains were ESBL producers. During the investigation of the antibiotic resistance profile of milk sample isolates carried out in Egypt, it was found that 86.11% of the strains were multi-resistant [40]. In the study of Elzhraa et al. [41], 44 *Salmonella* isolates were recovered from 280 Egyptian cheese samples. All isolates harbored virulence genes of *invA*, *stn*, and *hilA*. The highest resistance was found to be erythromycin and clindamycin (90.91%), as well as ceftazidime and cephalothin (84.09%). The majority of MDR isolates (79.55%) showed narrow spectrum (NS), had extended-spectrum (ES), and *AmpC-BLR* genes.

Table 1 summarizes the types of food products that were reported to contain antibiotic-resistant bacteria.

In Table 2, the most frequent and hazardous foodborne pathogenic bacteria are presented, which highlights their broadspectrum antibiotic resistance. They are among the top five foodborne pathogens that cause illnesses in the U.S. [42,43,44]. These were nontyphoidal *Salmonella* spp. (11%), *Clostridium perfringens* (10%), and *Campylobacter* spp. (9%). Those foodborne pathogenic bacteria among the top five foodborne pathogens reported to cause the highest number of death cases in the U.S. [42,43] were nontyphoidal *Salmonella* spp. (28%) and *Listeria monocytogenes* (19%). The top five confirmed foodborne infections, hospitalizations, and case fatalities were caused by *Campylobacter* spp., *Salmonella* spp., *Yersinia* spp., VTEC, and *Listeria monocytogenes* in the European Union and the U.S. [43].

**Table 1:** Antibiotic resistance of bacteria occurring in different types of food products.

Food Type		Bacteria	Antibiotics	Genes	Reference
**Vegetables, fruits**	Lettuce, romaine lettuce	*Staphylococcus aureus, Bacillus cereus, E. coli*, *Enterococcus* spp., *Aeromonas* spp., *Clostridium perfringens*, *Yersinia* spp., *Campylobacter* spp., *Salmonella enterica*, *Listeria* spp, *Klebsiella pneumoniae*	methicillin, macrolide, aminoglycoside, fosfomycin, lincosamide fluoroquinolone, β-lactam, rifampin, tetracycline, sulfonamides, vancomycin, lincosamides, and *type B streptogramin* (MLS*B*), oxytetracycline, carbapenem	*mecA, mdf(A), aph(3′)-Ia, fosA, lnu(A), lsa(A) and sal(A), oqxA, oqxB and qnrS1, mecA, blaTEM-116, blaACT-15, blaZ, blaLAP-2, blaOXY-1-3, tet(L), tet(M), BLA-1, BLA-2, sul1, str(A), erm(F), str(B), aad(A), int1, IncP oriT, IncQ repB, incW, int3, tet(A), tet(Q), tet(S), str(A), erm(B), blaOXA1, blaVIM, blaTEM* *tet(B), tet(C), tet(G), tet(L), blaOXA-48*	[45,46,47,48,49,50,51,52]
	Radish	*E. coli*, *Enterococcus* spp., *Aeromonas* spp., *Clostridium perfringens*, *Yersinia* spp., *Campylobacter* spp., *Salmonella enterica*, *Listeria*	aminoglycosides, beta-lactams, macrolides, sulfonamides, tetracyclines, vancomycin, lincosamides, and *type B streptogramin* (MLS*B*),	*sul1, str(A), erm(F), str(B), aad(A), int1, IncP oriT, IncQ oriV, int2, int3, tet(A), str(A), str(B), erm(B), erm(E), blaCTX-M, blaVIM, blaTEM*	[49,50]
	Carrot	*Staphylococcus aureus, E. coli*, *Enterococcus* spp., *Aeromonas* spp., *Clostridium perfringens*, *Yersinia* spp., *Campylobacter* spp., *Salmonella enterica*, *Listeria*	methicillin, macrolide, aminoglycoside, fosfomycin, lincosamide fluoroquinolone, β-lactam, sulfonamides, tetracyclines, vancomycin, lincosamides, and *type B streptogramin* (MLS*B*), colistin	*mecA, mdf(A), aph(3′)-Ia, fosA, lnu(A), lsa(A) and sal(A), oqxA, oqxB and qnrS1, mecA, blaTEM-116, blaACT-15, blaZ, blaLAP-2, blaOXY-1-3, sul1, str(A), erm(F), str(B), aad(A), int1, IncP oriT, IncQ oriV, int1, tet(A), tet(S), erm(B), erm(C), erm(E), blaVIM, blaTEM, mcr-1*	[45,49,50,53]
	Tomato, cherry tomato	*Staphylococcus aureus, E. coli*, *Clostridium perfringen, Yersinia* sp*., Campylobacter* sp.	methicillin, macrolide, aminoglycoside, fosfomycin, lincosamide fluoroquinolone, β-lactam, lincosamides, and *type B streptogramin* (MLS*B*), sulfonamide, tetracycline,	*mecA, mdf(A), aph(3′)-Ia, fosA, lnu(A), lsa(A) and sal(A), oqxA, oqxB and qnrS1, mecA, blaTEM-116, blaACT-15, blaZ, blaLAP-2, blaOXY-1-3 IncP oriT, incY, int2, int3, tet(A), tet(T) tet(S), aad(A), str(A), str(B), erm(B), erm(E), blaCTX-M, blaVIM, blaTEM*	[45,50]
	Pepper	*E. col*i, *Clostridium perfringen, Yersinia* sp*., Campylobacter* sp.	macrolides, lincosamides, and *type B streptogramin* (MLS*B*), aminoglycoside, sulfonamide, tetracycline, β-lactam	*nt3, tet(T), str(B), sul1, vat(B), blaOXAII*	[50]
	Cucumber	*Staphylococcus aureus, E. coli*, *Clostridium perfringen, Yersinia* sp*., Campylobacter* sp.	methicillin, macrolide, aminoglycoside, fosfomycin, lincosamide fluoroquinolone, β-lactam, lincosamides, and *type B streptogramin* (MLS*B*), sulfonamide, tetracycline,	*mecA, mdf(A), aph(3′)-Ia, fosA, lnu(A), lsa(A) and sal(A), oqxA, oqxB and qnrS1, mecA, blaTEM-116, blaACT-15, blaZ, blaLAP-2, blaOXY-1-3, IncP oriT, IncP trfA1, str(B), sul1, erm(B), blaOXAII*	[45,50]
	Spinach	*Pseudomonas teessidea*, *Morganella morganii*	cefotaxime, ceftazidime, carbapenem	*blaCTX-M-15, blaKPC*	[54,55]
	Garlic chives	*Bacillus cereus*	rifampin, tetracycline, β-lactam	*tet(L), tet(M), BLA-1, BLA-2*	[46,47,48]
	Perilla leaf	*Bacillus cereus*	rifampin, tetracycline, β-lactam	*tet(L), tet(M), BLA-1, BLA-2*	[46,47,48]
	Cabbage	*Staphylococcus aureus*	methicillin, macrolide, aminoglycoside, fosfomycin, lincosamide fluoroquinolone, β-lactam	*mecA, mdf(A), aph(3′)-Ia, fosA, lnu(A), lsa(A) and sal(A), oqxA, oqxB and qnrS1, mecA, blaTEM-116, blaACT-15, blaZ, blaLAP-2 and blaOXY-1-3*	[45]
	Watermelon, honeydew melon, peach, grape	*Staphylococcus aureus*	methicillin, macrolide, aminoglycoside, fosfomycin, lincosamide fluoroquinolone, β-lactam	*mecA, mdf(A), aph(3′)-Ia, fosA, lnu(A), lsa(A) and sal(A), oqxA, oqxB and qnrS1, mecA, blaTEM-116, blaACT-15, blaZ, blaLAP-2 and blaOXY-1-3*	[45]
	Orange	*Klebisiella pneumoniae*	colistin, polymyxin B, ampicillin	*bla, mcr-1, SHV-110*	[56]
	Apple	*E. coli*	aminoglycoside, colistin, polymyxin B, chloromycetin, sulfonamide, tetracycline, iclaprim	*mcr-1, aadA2, aadA1, floR, cmlA1, sul2, sul3, tetA, tetM, dfrA12, mdfA*	[56]
**Drinking water**		*Campylobacter* spp. *Enterococcus* spp. *Listeria* *Shigella* *Staphylococcus aureus* *Streptococcus pneumoniae* *Pseudomonas aeruginosa,*	erythromycin, aminoglycosides, amphenicols, quinolone, sulfonamides, tetracyclines, β-lactamase, vancomycin	*ermB* *aph(3′)-II* *cmlA, floR* *oqxB, qepA* *sul2* *tetO, tetQ, tetW* *blaTEM* *vanA*	[3,57,58,59,60,61,62,63,64]
**Meat/meat-products**	Hamburger broiler chicken, poultry	*E. coli*, *Salmonella* *Enterococcus* spp.	amoxicillin, penicillin, cephalexin	*blaTEM*	[65]
			erythromycin	*tet*M*, tet*L, *erm*B	[66]
			ciprofloxacin	*par*C, *gyr*A	[67,68]
**Dairy products**	Cheese	*Salmonella enterica*	trimethoprim/sulfamethoxazole, ciprofloxacin, cefoxitin, cefuroxime axetil, cefuroxime	*aac(6′), mdtK, cat_1, cat_4, golS, mdsA, mdsB, mdsC, rssB+, sdiA, ant(9)*	[69]
		*Enterococcus faecalis, E. faecium, E. gallinarum, E. avium, E. casseliflavis*	vancomycin, gentamicin, kanamycin, rifampin, tetracycline; erythromycin, lincomycin, linezolid, quinopristine/dalfopristine, chloramphenicol, streptomycin, ciprofloxacin	*tetM, ermB, cad, tetL, aph(3)IIIa, acc6-le-aph(2)-la*	[70]
		Gram-negative bacteria	cefepime, ertapenem gentamicin, ampicillin ampicillin, sulbactam, chloramphenicol, tetracycline, ciprofloxacin, ceftazidime, sulfamethoxazol, trimethoprim	*int 1, tet b, int 2, Shv, tet a, ctx—M, Tem, ctx- M15, oxa—48*	[71]
		*Salmonella* Typhimurium*, S.* Typhimurium*, S.* Infantis*, S.* Virchow*, S.* Tsevie*, S.* Rissen*, S.* Shubra*, S.* Anatum	ampicillin, amoxicillin, amoxycillin-clavulanic acid, cefazolin, cephalothin, cefoxitin, ceftazidime, cefepime, imipenem, meropenem, aztreonam, vancomycin, gentamicin, amikacin, neomycin, tetracycline, erythromycin, clindamycin, ciprofloxacin, sulfamethoxazole, trimethoprim/sulfamethoxazole	*blaOXA-1, blaOXA-2, blaTEM-1,* *blaCTX-M, blaCMY-1, blaCMY-2*	[41]
	Cheeses from Bovine, Ovine, and Caprine Milk	*Leuconostoc lactis, Leuconostoc mesenteroides, Lactococcus lactis, Lactococcus garviae, Enterococcus faecalis, Lacticaseibacillus plantarum, L. pentosus, L. delbrueckii, L. helveticus, L. brevis, L. casei, L. paracasei*	tetracycline, erythromycin, chloramphenicol	*tet(M,L,W), ermB, cat-TC*	[72]
	Raw milk and artisanal cheese	*Escherichia cvli*	amoxacillin—clavulanate, aztreonam, cefepime, ceftazidime, ceftriaxone, cefotaxime, meropenem, imipenem, cefoxitin, ampicillin, tetracycline, doxycycline	*blaTEM*	[73]
	Raw milk (Bovine)	*Escherichia cvli*	azithromycin, chloramphenicol, ceftriaxone, penicillin, gentamicin, amoxicillin, tetracycline, cephalexin	*bla*SHV*, bla*TEM	[37]
		*Listeria monocytogenes*		ND *	
		*Staphylococcus aureus*		*bla*Z*, mec*A	
	Raw milk (Bovine, Ovine, and Caprine)	*Staphylococcus aureus*	cefoxitin	*SCCmec- Iva*	[74]
	Mastitis milk (Bovine)	*Staphylococcus aureus*	cefoxitin, ampicillin, gentamicin, norfloxacin, streptomycin, ciprofloxacin, trimethoprim–Sulfamethoxazole, tetracycline, erythromycin, chloramphenicol	*bla*Z*, tet*M*, tet*K*, str*B*, msr*A*, erm*B*, erm*C	[75]
	Pasteurized milk	*Bacillus cereus, B. licheniformis, B. paralicheniformis, B. pumilus, B. safensis, B. Subtilis, B. toyonesis, B. invictae*	penicillin, ampicillin, tetracycline, trimethoprim- sulfamethoxazole	*tet*L	[76]

* ND: No data available.

**Table 2:** The top five foodborne pathogenic bacteria causing illness, hospitalization, and death and their antibiotic resistance properties.

Isolated Genera	Isolated Species/Serotype	Food Source	Resistance Phenotype	Resistance Genes	References
*Salmonella*	*enterica*/Typhimurium	poultry meat, eggs	amoxicillin-clavulanic acid, ampicillin, gentamicin, enrofloxacin, kanamycin, cefixime, cefepime, chloramphenicol, sulfamethoxazole/trimethoprim	*bla*_PSE-1_, *bla*_CMY-2_, *bla*_TEM_, *ampC*	[77]
	*enterica*/Infantis	food from animal origin	tetracycline	*tet(A)*	[78]
	*enterica*/Dublin	ground beef	ceftriaxone and tetracycline	*bla*_CMY-2_, *tet*(A)	[79]
	*enterica*/Derby and Typhimurium	pork, poultry	cefotaxime	*bla*_TEM_, *bla*_SHV_, *bla *_CTX-M_	[80]
	*enterica*/Heidelberg	pork chop, chicken breast	ampicillin, amoxicillin, clavulanic acid, cefoxitin, ceftiofur	*bla_CMY_*	[81]
	*enterica*/Kentucky	cow’s milk	nalidixic acid, ciprofloxacin, amoxicillin–clavulanic acid, cefotaxime	*bla_TEM_, ampC(FOX)*	[82]
	*enterica*/Anatum	cow’s milk	nalidixic acid, ciprofloxacin, ofloxacin	*qnrB*	
	*enterica*/Enteritidis	chicken meat	nalidixic acid, cefotaxime	*bla_TEM_, ampC(EBC)*	
*Campylobacter*	*jejuni, coli*	chicken, turkey, swine, cattle	tetracycline, quinolone	*tet* *(O), gyrA*	[83]
		chicken	cephalosporin, quinolone, fluoroquinolone		[84]
	*jejuni*	poultry	ciprofloxacin, nalidixic acid, tetracycline	ND *	[85]
*Clostridium*	*perfringens*	fish, shellfish	tetracycline, clindamycin, ampicillin, penicillin, ceftriaxone	ND	[86]
		duck	gentamicin, bacitracin, lincomycin, tetracycline	ND	[87]
		water	vancomycin, penicillin, erythromycin, tetracycline, trimethoprim, kasugamycin, bacitracin	*vanRG, vanRI, bla2, ermQ, tetB(P), dfrK, ksgA, bacA*	[88]
*Listeria*	*monocytogenes*	chicken meat	ceftriaxone, cefotetan, amoxicillin, amikacin, ertapenem, erythromycin, ciprofloxacin, trimethoprim	*sul*1, *sul*2	[89]
		food of animal origin	tetracycline	*tetM*	[90]
		freshly mixed sausage	cefoxitin, nalidixic acid, streptomycin, erythromycin, clindamycin, rifampicin, meropenem, tetracycline, trimethoprim–sulfamethoxazole	*tetM*	[91]
		juice	clindamycin, meropenem trimethoprim/sulfamethoxazole	*sul1*	[92]
*Yersinia*	*enterocolitica*	pork	neomycin, streptomycin, imipenem, sulfamethoxazole, vancomycin, nitroimidazole, amoxicillin, ampicillin, florfenicol, tiamulin, nalidixic acid	*emrD*, *yfhD*, *marC*	[93]
		meat	tetracycline, streptomycin, trimethoprim/sulfamethoxazole, cefazolin, chloramphenicol	*tetA, aph(6)-Id, aph(3″)-Ib, sul2*	[94]
		chicken meat	ampicilli, ticarcillin, cefoxitin	*blaA, blaB*	[95]
*Escherchia*	*coli*/verotoxin producing (VTEC)	meat	ampicillin, amoxicillin/clavulanate, caphalothin, streptomycin, tetracycline, nalidixic acid, trimethoprim/sulfamethoxazole	*bla_TEM_, strA, strB, tetB, sul2*	[96]
		milk	imipenem, meropenem, ampicillin, cephazolin, nalidixic acid, streptomycin, kanamycin, sulfamethoxazole/trimethoprim	*bla_VIM_, bla_TEM_*,	[97]
		meat	ampicillin, cephazolin, cefotaxime	*bla_TEM_, bla_CTX_*	
		beef	amoxicillin-clavulanic acid, ampicillin, aztreonam, chloramphenicol, ciprofloxacin, cefpodoxime, ceftriaxone, cefotetan, cefotaxime, cefoxitin, gentamicin, kanamycin, nalidixic acid, oxacillin, spectinomycin, streptomycin, sulfamethoxazole/trimethoprim, tetracycline	*bla_TEM-1_, qnrB, bla_CMY-2_, bla_CTX-M-3_, floR*	[98]
		chicken	amoxicillin-clavulanic acid, ampicillin, amoxicillin-clavulanic acid, ampicillin, aztreonam, chloramphenicol, ciprofloxacin, cefpodoxime, ceftriaxone, cefotetan, cefotaxime, cefoxitin, kanamycin, nalidixic acid, oxacillin, spectinomycin, streptomycin, sulfamethoxazole/trimethoprim, tetracycline	*bla_TEM-1_, bla_CTX-M-15_*	
		milk	amoxicillin-clavulanic acid, ampicillin, amoxicillin-clavulanic acid, ampicillin, aztreonam, chloramphenicol, cefotetan, ciprofloxacin, cefpodoxime, ceftriaxone, cefotaxime, cefoxitin, gentamicin	*bla_TEM-1_, qnrB, floR*	
		cheese	amoxicillin-clavulanic acid, ampicillin, amoxicillin-clavulanic acid, ampicillin, aztreonam, chloramphenicol, ciprofloxacin, cefotetan, ciprofloxacin, cefotaxime, cefoxitin, gentamicin, kanamycin, nalidixic acid, oxacillin, spectinomycin, streptomycin, sulfamethoxazole/trimethoprim, tetracycline	*bla_TEM-1_, qnrB, bla_CTX-M-15_, aac (6′)-Ib-cr*	

* ND: No data available.

### 2.1. Antibiotic Resistance and Tolerance: Adaptation Strategies

Bacteria have the capacity to adapt to their environment and develop mechanisms to survive and proliferate in the presence of antibiotics [99]. “*Adaptation is simply the process of evolution by natural selection*” [100]. Adaptation involves the progressive modification of microorganisms in a stressful environment to increase their tolerance [101]. Antibiotics are a significant source of stress for bacteria, prompting them to activate protective responses. Bacterial genome plasticity is imperative for the adaptation and response to environmental threats, including the presence of antibiotics [102]. An enhanced comprehension of bacterial stress responses and evolution indicates that, under certain conditions, the capacity of bacteria to withstand antibiotic therapy, either by transiently tolerating antibiotics or by evolving resistance, necessitates specific biochemical processes [103].

It is evident that several mechanisms can lead to resistance, and these have been the subject of detailed investigation [104]. These molecular mechanisms are categorized into classic and novel groups of resistance. Classic mechanisms encompass (i) antibiotic target modification or protection, (ii) antibiotic inactivation, (iii) increased efflux, or (iv) reduced uptake of antibiotics. Among the newly emerging antibiotic resistance mechanisms (v) the inactivation of bacterial metabolic enzymes, (vi) siderophore receptor mutation, (vii) formation of wall off antibiotics, and (viii) amplification of transposon in tandem array should be mentioned [105,106,107,108].

Moreover, a significant proportion of bacteria possess an inherent resistance to a wide range of antibiotics, including many commonly prescribed medications. Acquired resistance, on the other hand, is developed through gene mutations or via external genetic acquisition from nearby resistant organisms through horizontal gene transfer (HGT) [102].

Intrinsic resistance refers to the innate ability of species to resist a particular antibiotic agent due to their inherent structural and/or functional features (it is not transferable) [109]. It can be found in the genome of bacterial species and is independent of previous antibiotic exposure (antibiotic selective pressure) and HGT [110,111]. It is a stable, heritable trait specific to species or larger taxa. It may be linked to, e.g., the absence of a receptor for the antibiotic, a lack of affinity of the drug for the bacterial target, cell wall impermeability, or the presence of drug-degrading enzymes [112]. Intrinsic resistance includes, for example, the natural resistance of Gram-negative bacteria to vancomycin due to their cell wall structure (due to their large size and high molecular weight, these substances are unable to penetrate the outer membrane) [113].

The phenomenon of acquired resistance is achieved by the transfer of the genetic material conferring resistance. It is the result of mutations in the genetic material of the microorganism, or the transfer of the genetic material itself, which provides resistance via plasmids, bacteriophages, transposons, integrons, or other mobile genetic elements (MGEs), and is usually by conjugation, less often by transduction or transformation [1,114,115,116]. These MGEs can be horizontally transferred between different genera, even between pathogenic species. Acquired resistance develops as a result of selection pressure on the bacterial population [117].

Antibiotic resistance is a direct consequence of genetic alterations that are inherited by daughter cells; in contrast, antibiotic tolerance is an alternative strategy that enables survival in the presence of antibiotic doses that exceed MIC. The term antibiotic tolerance is frequently employed in scientific literature to denote the phenomenon of non-heritable antibiotic resistance. In the review by Grant and Hung [118], the term was defined as the reduced efficacy of antibiotics in the absence of genotypic resistance. In the presence of antibiotics, tolerant cells are unable to replicate, thereby maintaining MIC at its original level. These tolerant cells are killed at a slower rate than more sensitive cells, leading to an increased Minimum Duration for Killing (MDK) of the population [119].

The term “population-wide tolerance” is employed to denote all cells within a population that exhibit the tolerant phenotype. In contrast, “tolerance restricted to a subpopulation of cells” is referred to as “persistence” or “heterotolerance” [119]. In the context of persistent infections, it was observed that a population or subpopulation of bacteria may exhibit resistance to conventional antibiotics, potentially in a state of non-replicating or metabolically altered growth [118]. In such cases, bacteria adapt to the stresses imposed by the host environment by entering a different physiological state, such as a non-replicating or slowly replicating growth rate, or a small colony variant (SCV) phenotype. The size and composition of the persister subpopulation in bacterial communities are largely controlled by stress signaling pathways, such as the general stress response or the SOS response, in conjunction with the second messenger (p)ppGpp, which is almost always involved in persister formation [120].

Antibiotics may contribute to the occurrence of ARGs through multiple actions, including the exertion of selective stress to allow the accumulation of resistant strains, the promotion of the horizontal transfer of ARGs, and the facilitation of resistance mutations [121]. Stress-induced mutations, otherwise referred to as adaptive mutagenesis, were demonstrated to play a significant role in the progress of antibiotic resistance. Stress conditions caused by exposure to antibiotics are known to induce genotoxic stress in bacterial cells [122]. Stress proteins, also referred to as universal stress proteins (USPs), exist across a wide range of species and play a pivotal role in enabling organisms to withstand challenging environments [123].

Antibiotics require active cells to kill; however, persisters are a small subpopulation of cells that enter a dormant state and cease independent division. In the context of a bactericidal antibiotic treatment, regular cells perish, whereas persisters survive, thereby facilitating their tolerance [122,124]. The model developed by Kratz and Banerjee [125] demonstrates that cell death is seldom attributable to antibiotic levels that exceed the maximum physiological limit. Instead, survival is constrained by the inability to modify gene expression rapidly enough to transition to a less susceptible physiological state. Furthermore, bacteria often overexpress stress response genes, even at the cost of reduced growth, thereby conferring enhanced protection against further antibiotic exposure. This strategy is in contrast to those employed in different nutrient environments, in which bacteria allocate resources to maximize growth rate. This underscores a pivotal trade-off between the cellular capacity for growth and the ability to survive antibiotic exposure.

Given that both resistance and tolerance contribute significantly to the failure of antibiotic treatments, understanding the mechanisms of their evolution becomes imperative [126].

### 2.2. Effect of Different Stressors on Antibiotic Resistance of Foodborne Bacteria

During the production, distribution, and storage of food, as well as in the stomach and intestinal tract, microbial cells encounter several hurdles, such as suboptimal pH, suboptimal temperature or salt concentration, the impact of bile salts, and the presence of antimicrobial compounds such as bacteriocins and disinfectant residues [127,128,129].

These stress factors can alter microbial cells, affecting cellular processes and resistance. The modification in resistance may result from a combination of stress response and molecular mechanisms of resistance to antibiotics [130].

Various publications suggest a plausible correlation between stress adaptation in foodborne bacteria and the development of antibiotic resistance [131,132]. Alternative sigma factor (σB) may play a role in stress adaptation, which is a contributing factor in the expression of virulence and stress response genes. Additionally, two-component signaling systems (2CSTS) were demonstrated to play a role in the innate cephalosporin resistance of *L. monocytogenes* [133]. It was observed that the adaptive response in bacteria to various food-associated stresses provides cross-protection to antibiotics, which may accelerate the dissemination/spread of antibiotic resistance in the food chain.

The stresses that arise in the food chain also affect the antibiotic resistance of starter cultures by inducing changes in gene expression [134]. Limited research exists concerning the effect of stress on the antibiotic resistance of lactic acid bacteria. Amund and colleagues [135] conducted a study on the impact of acid and bile stresses on *Lactobacillus*. Their findings revealed that the effects of the stressors were varied; an increase in resistance was observed in some cases and a decrease in others depending on the type of stress, bacterial species or strain, and the type of antibiotic. The research of Natt and Garcha [136] demonstrated that *Lactobacillus acidophilus* cultures, which adapted to acidic stress conditions, exhibited higher resistance to antibiotics in comparison to their optimal pH counterparts. The strain selected for analysis was sensitive to all antibiotics used in the experiment, i.e., ampicillin, streptomycin, vancomycin, penicillin, chloramphenicol, and tetracycline, except erythromycin. The authors showed that after exposure to the stressor, the test strain showed higher resistance to all other antibiotics except tetracycline and chloramphenicol.

Casado Muñoz et al. [137] observed an increase in the MIC of ampicillin, chloramphenicol, ciprofloxacin, and tetracycline in *Leuconostoc pseudomesenteroides* and *Lactiplantibacillus pentosus* (formerly *Lactobacillus pentosus*) due to exposure to physicochemical stress, including antimicrobial agents, UV radiation, and chemicals such as isopropyl-b-D-thiogalactopyranoside, NaCl, and ethanol.

In their examination of the impact of ionizing radiation in 2024, Kovács et al. [138] demonstrated that the genome of *S. aureus*, which is also a significant concern from the perspective of food safety, is modified by gamma radiation, resulting in the degradation of the *mecA* gene that encodes β-lactamase resistance and the loss of its resistance to oxacillin.

The induction of a heat shock response was also demonstrated to result in macrolide resistance in *Lactococcus lactis* [139]. The effect was explained by the fact that the observed changes in antibiotic resistance levels due to the stress factor may result from the triggering of the stress response. The phenomenon can be observed when antibiotic resistance genes and genes induced during a stress factor are located on the same operon and are simultaneously induced.

## 3. Antibiotic Resistance in Traditional Foods

### 3.1. Antibiotic Resistance in Vegetables and Fruits

The consumption of fresh vegetables and fruit is essential for human health and has increased in recent decades [140,141,142]. They are often consumed raw, without any processing steps [143,144]. The number of foodborne illnesses associated with vegetables and fruits has increased in recent decades. This is due to the susceptibility of these plants to microbial contamination through a number of potential pathways, such as the use of animal manure, contaminated irrigation water, irrigation with wastewater, and so on [49,145,146,147,148,149]. Therefore, the safety of edible plants is dependent upon the quality and safety of the water and soil in which they are cultivated. In some cases, the products may be safe, whereas in others, they may pose a microbiological hazard [150].

Previous studies have shown that plant-based products, particularly when consumed raw, are identified as a potential vector for the transmission of pathogens, from the environment to humans [151,152,153,154,155,156,157]. These pathogens include ARB and ARGs, which pose a significant public health threat [140,158,159,160,161,162]. The rationale behind this phenomenon pertains to the transportation of unmetabolized antibiotics from hospital wastewater to wastewater treatment plants, where the removal of antibiotics is incomplete and ARGs are eventually released into the natural aquatic environment [163]. Antibiotics are thus released into surface waters, where antibiotic concentrations in the range of micrograms per liter have been reported [164,165]. Besides hospital wastewater, household wastewater also plays an important role in the spread of antibiotic resistance. A significant proportion of antibiotics is used in people’s homes and thus enters the sewage treatment system through domestic wastewater. Urban wastewater treatment plants are increasingly acknowledged as critical sources of ARB and ARGs released into the environment. These facilities process sewage originating from a variety of sources, thereby amalgamating bacterial populations from diverse ecological niches. This amalgamation fosters interactions among bacteria and facilitates HGT [166].

Although antibiotic usage in plants has constituted less than 0.5% of the total antibiotic use [167], the recent approval of streptomycin and oxytetracycline for the prevention of citrus diseases (citrus canker and citrus greening disease) has resulted in an 18-fold increase in the agricultural use of these antibiotics [168]. The aforementioned infection pathways not only permit the transmission of pathogenic bacteria to plant foods but also help to increase the abundance of ARGs and facilitate the entry of these ARGs, especially into fresh vegetables [169,170]. The utilization of manure-derived fertilizers, poor quality irrigation water, the recycling of containers for the transportation of agricultural products, and other factors are among the key factors of these entry routes [169,170,171,172]. Therefore, it can be reasonably deduced that the ingestion of fresh vegetables may significantly contribute to the dissemination of antibiotic resistance in humans.

The first report was published in 2014 on extended-spectrum β-lactamase (ESBL)-producing isolates from vegetables and fruits originating from the Netherlands [173]. In their study, the *blaFONA-5* gene was identified in *Serratia fonticola*. The *blaRAHN-1* and *blaRAHN-2* genes were detected in *Rahnella aquatilis* strains. Since this first report, the presence of ESBL-producing Gram-negative bacteria in fresh vegetables and fruits has been documented in numerous countries worldwide [140,174,175,176,177,178]. Salmanov et al. [179] found that the overall proportion of ESBL-producing *Enterobacteriaceae* was 36.8% from fresh vegetables available in the Kyiv city markets (Ukraine). ESBL-producing pathogens were found in fresh produce in Japan [178]. A variant of the *shv* gene (*blaSHV-110*) was identified by Yang et al. [56] in *Klebisiella pneumoniae* isolates from orange samples obtained from Chinese markets. Similarly, Trocado et al. [180] reported the presence of the same gene in three isolates from an orange juice sample. A German study [181] reported the isolation of seven ESBL-producing *E. coli* isolates from fresh vegetables. The isolates were positive for *blaCTX-M-14*, *blaCTX-M-15*, *blaCTX-M-65*, *blaCTX-M-125*, and *blaCTX-M-2* genes. Mesbah et al. [182] documented the occurrence of multidrug-resistant *Klebsiella pneumoniae* isolates including ESBL genes in fresh fruits and vegetables sold in Algerian markets. The study of Sun et al. [183] from China revealed that of the 48 *E. coli* isolates, 28 (58.3%) were identified as ESBL-producing. Of these, 4 (66.7%, 4/6) were collected from soil, 6 (40.0%, 6/15) from vegetables, and 18 (66.7%, 18/27) from irrigation water. Chinese resistance surveillance data conducted in 2021 indicated a significant increase in the proportion of ESBL-producing *E. coli*, reaching 52.6% [184]. A high prevalence (83.3%; 20/24) of ESBL-producing strains from fresh vegetables and RTE salads [185] was published concerning Italian fresh produce. In contrast, there was a much lower frequency of ESBL-positive isolates in products tested in South Korea [176]. Among the analyzed 1324 raw vegetable samples, 0.83% (11/1324) were ESBL-positive *E. coli* strains. Kayode and Okoh [177] published a paper on variants of ESBL resistance in *L. monocytogenes* strains from fruits and vegetables in South Africa besides *L. monocytogenes* isolates, that encoded resistance to a range of other antibiotics, including tetracyclines, sulfonamides, phenicols, and aminoglycosides.

The first report on *mcr-1*-producing *E. coli* isolated from fresh produce was published in Switzerland in 2016 from ready-to-eat vegetables grown in Thailand and Vietnam. The two isolates were found to carry the *mcr-1* gene together with the *blaCTX-M-55* and *blaCTX-M-65* genes [186]. Since the first isolation, *mcr* gene-producing Gram-negative bacteria have been reported worldwide [53,56,160,187,188]. In South Korea, Oh et al. [189] identified the *mcr-1* gene in *E. coli* isolates from 0.076% (1/1324) of the investigated vegetable samples. The presence of the gene-encoding *mcr-1* plasmid-mediated colistin resistance was reported in an *E. coli* isolated from lettuce grown and marketed in Portugal [187]. In a study conducted by Liu et al. [53], the authors analyzed *mcr* genes in 528 vegetable samples sourced from 53 supermarkets or farmers’ markets across 23 cities in nine provinces in China. Twenty-three *E. coli* and one *Enterobacter cloacae mcr-1*-positive isolate were obtained, which were derived from 19 (3.6%) vegetable samples. Fruit samples from China have also shown the presence of *mcr* genes [56]. This study conducted an examination of 133 fruit samples to determine the presence of various MCR variants (*mcr-1* to *mcr-8*). This finding revealed the first identification of *mcr-1*-carrying *E. coli* and *Klebsiella pneumoniae* in market retail fruits in Guangzhou, China. In Japan, 308 colistin-resistant isolates were detected in 200 fresh vegetable samples [188]. Despite the absence of positive *mcr-1* to *mcr-8* genes among the isolates, one *Enterobacter cloacae* strain and a *Raoultella ornithinolytica* were identified as positive for the *mcr-9.1* allele. The first Algerian report of the detection of the *mcr-1* gene from vegetables was published by Chelaghma et al. [190]. From the analyzed 400 fresh vegetable samples, the *mcr-1* gene was detected in only two *E. coli* isolates.

The first publication of carbapenemase-producing *Klebsiella variicola* from fresh vegetable samples was published in 2015 [191]. The isolated strain was positive for the *blaOXA-181* gene. It was isolated from a coriander sample from Thailand/Vietnam. Carbapenem resistance was detected in 35.3% of *Pseudomonas aeruginosa* and 66.8% of *Acinetibacter* spp. isolates from fresh vegetables sold at a retail market in Kyiv (Ukraine) [179]. Carbapenemase-producing bacteria were observed in 2.4% of the vegetables analyzed in Romania [55]. Carbapenemase production was detected in 4 (0.47%) of the 856 bacterial isolates from vegetable samples. Carbapenem-resistant *Klebsiella pneumoniae* was detected from leafy vegetables from Gondar, Ethiopia [192]. All isolated *K. pneumoniae* strains were resistant to the carbapenem drugs. Among the carbapenems genes, *NDM-1*, *blaOXA48*, *blaVIM*, and *blaIMP* were found. Nketiah et al. [193] examined the carbapenem resistance in *E. coli* from ready-to-eat fresh-cut fruits in Accra, Ghana. A total of 5.9% of the 34 *E. coli* isolates exhibited resistance to carbapenem and contained the carbapenemase gene *blaIMP*.

It can be concluded that the consumption of fresh vegetables and fruits may significantly contribute to the dissemination of antibiotic resistance in humans.

### 3.2. Antibiotic Resistance of Foodborne Pathogenic Bacteria in Meat

In recent years, there has been an observed increase in antibiotic resistance among pathogens present in meat and meat products. This phenomenon can be attributed to the excessive and unregulated use of antibiotics in the food production process. Pathogenic bacteria present in meat such as *Salmonella, Campylobacter, E. coli*, and *L. monocytogenes* have demonstrated resistance to important antibiotics such as tetracyclines and sulfonamides, which are essential for effective treatment [194]. *Enterococcus* species, *Enterococcus faecium* and *Enterococcus faecalis*, showed resistance to antibiotics such as vancomycin [195,196].

A substantial number of studies have demonstrated the role of meat in the dissemination of antibiotic resistance in the world of food safety. Rajaei et al. [65] investigated antibiotic resistance of pathogenic bacteria (isolated from raw kebab and hamburger samples) in Iran. *E. coli* had the highest prevalence, with 70% of kebab and 48% of hamburger samples positive for this bacterium. The study showed high resistance to antibiotics such as amoxicillin, penicillin, and cephalexin, with the *blaTEM* gene as the most common resistance gene in *E. coli* and *Salmonella* isolates. In a similar study, *Campylobacter*, *E. coli*, *Listeria*, and *Salmonella* were identified in various samples taken from food desert retail outlets in Virginia, USA [197]. Resistance to ampicillin and tetracycline was predominant, with higher contamination rates in smaller private markets than in supermarkets. This fact underlines the importance of food safety regulations in different retail settings [197]. In a study, Liu et al. [198] focused on retail beef and mutton, and pathogens that have mobile antimicrobial resistance genes. They identified *Klebsiella* spp. and *Staphylococcus* spp. as the dominant species in the samples, with resistance to antibiotics such as tetracyclines. The presence of the extended-spectrum β-lactamase (ESBL) gene was also detected in the *Klebsiella pneumoniae* species, which is an indicator of the possible spread of resistance through MGEs [198].

Research on antibiotic resistance was conducted on different meat samples. Gutema et al. [199] investigated *E. coli* O157 in cattle, beef, and humans in Ethiopia. The study revealed that *E. coli* was prevalent in cattle (7.1%) and beef (6.3%), with genetically similar strains detected in all samples. This finding suggests the potential for transmission through the consumption of beef. The majority of *E. coli* samples that are found to be positive for the *stx2* gene, which is associated with the production of Shiga toxin, have the potential to cause severe illness in humans [199]. Obaidat [200] investigated the prevalence of antibiotic resistance in *L. monocytogenes*, *Salmonella enterica*, and *E. coli* O157 in imported beef cattle in Jordan. This study showed a high prevalence of resistance to multiple antibiotics in *Salmonella* and *E. coli*, with resistance to antibiotics such as nalidixic acid, ciprofloxacin, and ceftriaxone [200].

The problem of antibiotic resistance is also present in poultry. Zamil et al. [201] found high levels of resistance in *Salmonella* isolated from chicken hatcheries, while Li et al. [202] detected resistance to carbapenems and colistin in *E. coli* strains from Chinese poultry farms. These findings coincide with global trends in AR, which highlight the need for better and stronger regulation of antibiotics in the meat industry [203,204]. Rehman et al. [66] studied the distribution of antibiotic resistance in *Enterococcus* species in poultry treated with different antibiotics. They found that *Enterococcus faecium* and *Enterococcus faecalis* are the most common species that are resistant to ciprofloxacin, macrolides, penicillin, and tetracycline. Similarly, Yu et al. [67] investigated the molecular characteristics of *Enterococcus faecalis* isolated from chicken in China. Their findings showed different levels of resistance to antibiotics such as erythromycin (96.72%) and tetracycline (96.72%), while resistance to vancomycin was quite low (8.2%). The research also found many resistance genes in the isolates, such as *ermB*, *tetM*, and *tetL* [67].

These studies highlight the need for alternatives such as probiotics, bacteriophages, and vaccines to reduce the use of antibiotics in food-producing animals [205]. These alternatives, together with strict safety laws, are very important in reducing the spread of AR in the meat industry [206]. We can conclude that pathogens related to meat and meat products, such as *Salmonella*, *Campylobacter*, *E. coli*, *Enterococcus faecalis*, and *L. monocytogenes*, represent one of the greatest dangers in public health. Excessive use of antibiotics in meat has resulted in the growth of MDR bacteria, which are dangerous for both humans and animals.

### 3.3. Antibiotic Resistance in Dairy/Fermented Foods

#### Antibiotic Resistance of LAB in Fermented Dairy Products

Lactic acid bacteria are Gram-positive, non-spore forming, catalase-negative, acid-tolerant, aerotolerant, usually non-motile cocci or rods (*Lactiplantibacillus*, *Lactobacillus*, *Enterococcus*, *Streptococcus*, *Leuconostoc*, *Weisella*, *Pediococcus*, *Lactococcus*, etc.). LAB constitute the most crucial microorganisms in fermented foods, such as yogurts, cheeses, and salami. The current trends in the food industry and the growing demand for healthy foods have led to the development of fermented dairy foods that provide health-promoting antimicrobial metabolites, prebiotic substances, diverse probiotic bacteria with immune system stimulating effects, unique flavors, and nutritional benefits shaped by regional ingredients and processing methods [207,208]. Different LAB and bifidobacteria are used as starter cultures and probiotics to create fermented functional foods and remain active in the product, interacting with microbiota and intestinal wall cells during transit [209]. When employed as protective cultures, the antimicrobial metabolites they produce (such as bacteriocins, organic acids, and H_2_O_2_) are utilized for their effectiveness against the spoilage-causing and pathogenic microorganisms (such as *L. monocytogenes, Clostridium*, and *Bacillus* species) present in food [210,211,212,213]. As bioprotective cultures, they are regarded as an alternative to antibiotics in animal production, due to their impact on pH and ability to act as antimicrobial agents in inhibiting zoonotic pathogens [214,215].

While LAB are recognized as safe and widely used in food and fermented products, they have the potential to harbor antibiotic-resistant genes, colonize the intestine, and facilitate horizontal transferring of these genes to commensal and pathogenic bacteria in the food chain; therefore, they are considered as “reservoirs” of ARGs [216,217,218,219,220].

Fermented foods contain significant amounts of LAB, leading to their high consumption by consumers. LAB in the human gut have the potential to share genetic components with other nearby microbes [221,222]. Physical proximity of bacteria in the gut invariably raises the likelihood of HGT [223,224,225]. This probability is elevated even further when antibiotic resistance genes are on MGEs, such as on plasmids [218]. Thus, LAB can act as a source of environmental antibiotic resistance genes [224,226]. Wild-type LAB strains and commercial starters both contribute to ARG dissemination throughout the food chain. The latter’s impact was initially documented by Luo et al. in 2005 [227]. Since then, multiple publications have demonstrated that GRAS strains, such as those used in food starters, are capable of acquiring antibiotic resistance determinants and transferring them to other strains [228]. Jacobsen et al. [229] reported the in vivo transfer of wild-type antibiotic resistance plasmids from *Lactiplantibacillus plantarum* (formerly *Lactobacillus plantarum)*, which were isolated from fermented dry sausage, to *Enterococcus faecalis* into JH2-2, a natural inhabitant of the human gut. The transfer of resistance genes between commercial strains and commensal gut bacteria in vitro and in vivo was confirmed by other studies [5,226,228,230,231,232]. Nawaz et al. [224] presented evidence of the transfer of the erythromycin resistance gene from *Lactiplantibacillus plantarum* (formerly *Lactobacillus plantarum)* and *Lactiplantibacillus brevis* (formerly *Lactobacillus brevis*) to *Enterococcus faecalis*. From this, it can be inferred that fermented dairy products can also serve as a possible medium for antibiotic-resistant bacteria to enter into the human body.

Recently, it was demonstrated that in addition to commensal LAB strains showing single or multiple antibiotic resistance, resistance genes were also reported in probiotic GRAS strains [219].

Phenotypic characterization of AR patterns among LAB strains derived from traditional fermented foods reveals significant variability, depending on LAB species and antimicrobial agents [228,233]. Antibiotic resistance in wild and commercial LAB is observed worldwide. Resistance to various antibiotics, including ampicillin, vancomycin, erythromycin, tetracycline, chloramphenicol, and ciprofloxacin, was demonstrated in *Streptococcus thermophilus* strains that are currently used as starters in the dairy industry [224,234,235,236]. According to the review of Nunziata et al. [237], resistance to gentamicin, kanamycin, chloramphenicol, tetracycline, and erythromycin is most commonly found in starter cultures and industrially important strains. Several studies have shown that the prevalence of antibiotic-resistant LAB isolates is greater than 50%. In more detail, the analysis of *Lactobacillus* spp. demonstrated a 58% resistance to vancomycin, while *Bifidobacterium* spp. exhibited 60% resistance to vancomycin, whereas all tested strains of *Enterococcus* spp. showed 100% resistance to vancomycin, erythromycin, rifampin, and ciprofloxacin [238]. Vancomycin resistance in enterococci poses a major challenge in the treatment of infections as it signifies the absence of effective antibiotic treatment for multi-resistant enterococci infections [239,240]. Previous research has documented the identification of *Enterococcus* strains that are resistant to antibiotics and carry virulence factors in cheeses [241,242,243].

Nowadays, raw milk consumption has become a common practice in developed countries [244,245] due to its perceived health benefits. It is noteworthy that antibiotic residues were found to be present in unpasteurized milk samples (23.8%) from the same region more frequently than in pasteurized samples (6.8%). Cheeses produced from unpasteurized milk are favored by some consumers for their more diverse flavors and aromas. However, such products may contain harmful foodborne pathogens like staphylococci, *L. monocytogenes*, and *E. coli* [246,247]. Alexa et al. [248] found high levels of multi-resistant *Lactococcus lactis* in cheese samples made of raw milk, along with relatively elevated concentrations of *E. coli* and *Salmonella enterica* subsp. *enterica*. Antibiotic-resistant bacteria were also found in various fermented milk products. In a study that investigated *Lactobacillus* isolated from traditional dairy products, 19 vancomycin-resistant, 10 ciprofloxacin-resistant, and 1 tetracycline-resistant bacteria were detected in fermented yak, cow, and mare milk [249]

A recently published study has confirmed that LAB strains from fermented foods and human sources exhibit significant phenotypic resistance to cephalosporins, aminoglycosides, quinolones, and glycopeptides [250], regardless of their origin. All strains of lactic acid bacteria isolated from Brazilian dairy products demonstrated resistance to oxacillin and sulfa trimethoprim [250]. During a study of fermented food products (including meat and dairy) in Turkey, the research uncovered a high prevalence of vancomycin-resistant lactic acid bacteria (VRLAB) with an existing resistance of 57.45%, 53.19%, and 44.68% to ciprofloxacin, norfloxacin, and teicoplanin, respectively [251]. Haryani et al. [252] demonstrated a prevalence of 92% for MDR LAB isolates in Malaysian fermented food. All *Pediococcus* and *Weissella* isolates and 53.85% of the *Enterococcus* derived from fermented dairy and meat products exhibited multiple AR [251]. *Bifidobacterium* species exhibited resistance to vancomycin at a rate of 60% [238], along with tetracycline and ciprofloxacin [228] and chloramphenicol [237]. Various literature [253,254,255] has demonstrated that probiotics such as *Lactobacillus* (*Lacticaseibacillus paracasei* (formarly *Lactobacillus paracasei*)*, Ligilactobacillus salivarius (*formerly *Lactobacillus salivarius*), and *Lactiplantibacillus plantarum* (formerly *Lactobacillus plantarum*)), *Enterococcus*, *Lactococcus lactis*, and *Bifidobacterium* displayed complete intrinsic resistance to last-resort antibiotic colistin [1], as well as kanamycin, neomycin, ciprofloxacin, vancomycin, gentamicin, and streptomycin, with specific resistance patterns observed in various species retrieved from fermented milk products [233] and dairy environments [217,250,256]. Meanwhile, *Lactobacillus bulgaricus*, *L. acidophilus*, and *S. thermophilus* showed different degrees of susceptibility to vancomycin, suggesting the inherent susceptibility of some LAB strains to this glycopeptide antibiotic [224,257]. Furthermore, a broad range of LAB displayed sensitivity to ampicillin, clindamycin, erythromycin, cefsulodin, penicillin G, and rifampicin, reflecting that these antimicrobial agents are still effective for targeting lactobacilli [224]. Despite this, a concerning trend of acquired resistance to penicillin, erythromycin, clindamycin, and tetracycline was observed in multiple species of LAB from diverse sources, such as fermented milk [238,258], probiotics or fermented foods [254,259], and human intestine [219]. The emergence of such resistant strains outlines the dynamic nature of AR among LAB, posing potential risks for both food safety and public health.

Many AR genes in *Bifidobacterium*, *Lactobacillus*, *Lactococcus*, *Leuconostoc*, *Pediococcus*, and *Streptococcus thermophilus* were acquired through conjugative plasmids [260]. The characterization of AR genes in fermentative bacteria is often incomplete and represents a significant risk, as many of these genes may remain undetected or be novel. Fortunately, breakthroughs in genome sequencing and metagenomic analysis are helping to reveal this concealed realm, allowing us to better protect our food by uncovering the actual profile of AR genes in fermented products [261]. Almost 89% (8/9) of LAB strains derived from yogurt and cheese commercially available in Tianjin showed resistance to at least one antibiotic and were positive for *van*, *aph*, and *aadA2* resistance genes [222]. Moreover, several resistance genes, including *tet(M)*, *strA*, *strB*, *sul1*, *sul2*, *aac(6′)*, *aph(2″)*, *aph(3″)-II*, and *aph(3″)-III*, were detected by Li et al. [258] in 87 LAB strains isolated from fermented milk products in China. The results show that these bacteria do not transmit genes but pose a threat in spreading antibiotic resistance. However, Thumu and Halami [230] declared that three strains of *Lactobacillus* originally isolated from chicken meat showed the ability to transfer *erm(B)* and *tet(M)* AR genes to pathogenic bacteria in vivo (using rats), in vitro, and during the food fermentation process.

Tetracycline resistance in lactobacilli involves 11 transferable AR genes, some of which, like *tet(K)* and *tet(L)*, are plasmid-coded, while others, like *tet(M)* and *tet(O)*, are both plasmid and chromosomally coded [1]. A recent analysis of 47 shotgun sequencing datasets from various probiotic samples reported over 70 AR genes, including those related to rifampicin, tetracycline, and extended-spectrum β-lactamase (ESBL) resistance. Alarmingly, many of these AR genes were associated with MGEs, plasmids, or phages, simplifying the transfer risk to human gut microbiota, and raising significant public health concerns [262]. In addition, Fatahi-Bafghi et al. [263] analyzed 126 whole genomes of probiotic bacteria for AR genes. The results demonstrated that tetracycline (*tet*) and erythromycin (*erm*) resistance genes were prevalent, particularly in *Bifidobacterium* and *Lactobacillus*. These data raise critical public health concerns and highlight the need for incessant screening of probiotics and fermented foods for AR to ensure food safety.

Scientific evidence has prompted EFSA to establish criteria for evaluating the safety of microorganisms used in food production [264]. Furthermore, the EFSA FEEDAP Panel has recently developed a microbiological cut-off value (mg/L) that can distinguish between resistant and susceptible LAB and *Bifidobacterium* strains [225]. The EFSA-Feedap [225] guidelines on the characterization of microorganisms used as feed additives or production organisms provide further insight. Moreover, both EFSA and WHO have advised the exclusion of bacterial strains with MGEs containing ARGs from use in feed, food fermentation, and probiotics to prevent the transfer of resistant genes through HGT from non-pathogenic to pathogenic bacteria, thereby increasing antibiotic resistance in humans and posing a threat to public health. Therefore, from the perspective of food safety, the phenotypic AR pattern is a crucial criterion for selecting probiotic strains for the preparation of nonhazardous fermented dairy foods. Strains exhibiting desirable AR profiles ensure that they do not contribute to the potential transfer and spread of AR genes while maintaining the probiotic benefits for fermentation processes, gut health, and food preservation [265,266]. Research suggests that countries without or with recently implemented antibiotic restrictions have a higher incidence of isolation of resistant strains. Conversely, in European nations, bacteria displaying phenotypic resistance were predominantly observed in handicraft products that were constrained to a specific geographic region [237].

**Figure 1:**
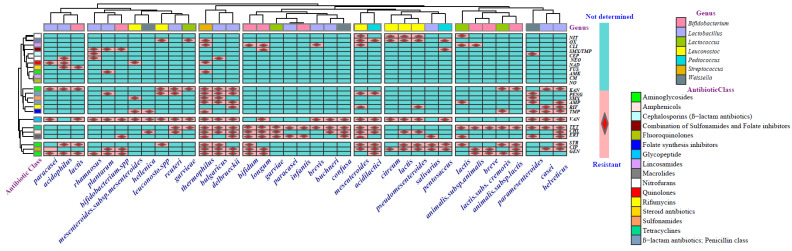
A complex heatmap with hierarchical clustering that depicts the phenotypic antimicrobial resistance profiles of seven LAB genera. The *X*-axis displays LAB serovars (n = 37), while the *Z*-axis represents the panel of antibiotic disks tested (n = 24). The cells with red diamonds indicate resistance and turquoise cells signify either susceptibility or an unidentified characteristic [5,216,219,228,256,267,268,269,270,271,272,273,274,275,276,277,278,279,280,281,282,283,284,285,286,287,288,289,290].

**Figure 2:**
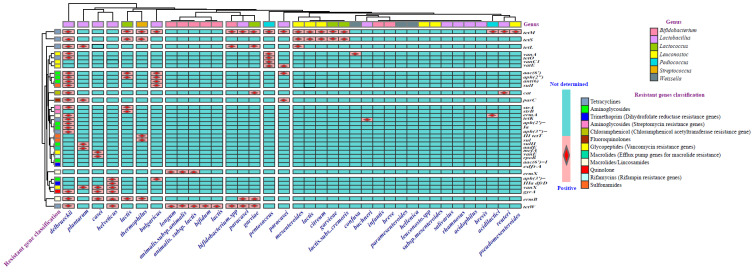
A complex heatmap with hierarchical clustering that illustrates the existence pattern of twelve antibiotic class resistance genes in seven LAB genera. The *X*-axis displays LAB serovars (37), while the *Z*-axis represents the panel of M-PCR amplified resistant genes (n = 35). The cells with red diamonds indicate existence and turquoise cells signify either gene absence or an unidentified characteristic [5,216,219,228,256,267,268,269,270,271,272,273,274,275,276,277,278,279,280,281,282,283,284,285,286,287,288,289,290,291].

Based on the information presented, it can be concluded that though the major starter culture companies are continuously working to screen commercial cultures for resistance and eliminating antibiotic-resistant strains from their product lines [237], the starter cultures with the potential to transfer antibiotic resistance are currently used in industrial dairy products in certain regions of the world. In Figure 1 and Figure 2, the phenotypic antimicrobial resistance of seven LAB genera and the existence pattern of twelve antibiotic class resistance genes in seven LAB genera are shown.

## 4. Antibiotic Resistance in Drinking Water

In recent years, studies have indicated that the drinking water treatment process is not fully effective in eliminating all microorganisms. This has resulted in the resurgence of disinfectant-resistant bacteria within drinking water distribution systems (DWDSs) [292,293]. Research has demonstrated that the use of chlorine as a disinfectant can promote the selection of ARB, which in turn increases the prevalence of ARGs in drinking water systems. This situation presents a significant public health concern on a global scale [292,294,295,296,297,298,299].

The presence of biofilms is a common phenomenon, particularly in decentralized wastewater treatment systems (DWTSs). A biofilm can be defined as a biologically active matrix that is attached to the cell surface and the extracellular substances (EPSs—extracellular polymeric substances) that are released by the cells. Furthermore, the biofilm functions as a bacterial community, whereby a multitude of harmful substances, including residues from water treatment and pathogens, can adhere to it and to surfaces in contact with the biofilm [300]. This has rendered biofilms a principal conduit for the dissemination of antibiotics [291]. In these biofilms, bacteria reside in close proximity to one another, forming a high-density cellular environment. This may facilitate the spread of antibiotic resistance, as HGT is more common in dense bacterial populations. Furthermore, extracellular polymeric substances (EPSs), within which biofilm cells are embedded, afford protection to microorganisms against deleterious agents, including disinfectants and mechanical impacts [107,292,301,302,303,304,305]. Environmental changes resulting from stress enable cells to adapt to novel adverse conditions, thereby contributing to the emergence of new bacterial phenotypes. Bacterial genomes may harbor mutations or genes that confer a survival advantage in the presence of antimicrobial agents. Antibiotic-susceptible bacteria can acquire resistance through de novo gene mutations or by adopting resistance genes from other bacteria [3,306].

Several bacteria were previously identified in DWDSs, and a significant proportion of these were found to exhibit resistance to antibiotics. The bacterial species identified include *Staphylococcus*, *Enterococcus*, *Pseudomonas*, *Ralstonia*, *Mycobacteria*, *Clostridium* species, and the *Enterobacteriaceae* family, as well as Gram-negative bacteria. Additionally, some pathogenic or opportunistic pathogenic bacteria showing resistance in biofilms against different antibiotics were identified in drinking water [3,58,59,60].

Sulfonamides are recommended for the treatment of many *Nocardia* infections, especially in hospital settings. Tetracyclines are also among the most commonly used antibiotics in both human health and veterinary medicine, particularly for the treatment of bacterial infections in food-producing animals [58,60,307,308].

The persistence of ARB and ARGs in drinking water systems underscores the urgent need for stringent monitoring, regulatory measures, and innovative treatment solutions.

## 5. Antibiotic Resistance in Novel Foods

According to the Regulation (EU) 2015/2283 [309] “‘novel food’ means any food that was not used for human consumption to a significant degree within the Union before 15 May 1997, irrespective of the dates of accession of Member States to the Union”. Novel foods must be classified in one of ten specified categories according to the regulations (Table 3). Genetically modified foods, food enzymes, food additives, food flavorings, and extraction solvents are not encompassed within the scope of this regulation [310].

The present chapter concentrates on the potential for the transmission of antibiotic resistance in food consisting of microalgae and insects.

### 5.1. Antibiotic Resistance Gene Migration Between Microalgae and Bacteria

Microalgae constitute a diverse group of single-celled photosynthetic organisms, encompassing both prokaryotic (*Cyanobacteria*—blue-green algae) and eukaryotic (e.g., Chlorophyceae—green algae; Porphyridiophyceae—red algae; Bacillariophyceae—Diatoms) species [315,316,317]. Microalgae are regarded as future food and feed due to their high nutritional values (protein, polyunsaturated fatty acids, and vitamin content) [318,319,320], the capacity for rapid proliferation in a diverse range of habitats under photoautotrophic conditions, their simpler genetic manipulation, and their more straightforward scale up processes [321,322].

The cyanobacterium *Arthrospira platensis* (also known as Spirulina) and the eucaryotic *Chlorella* species have been used as food sources or ingredients in several countries all over the world for a long time [323,324]. Therefore, these microalgae are not considered as novel foods [311]. *Arthrospira platensis* and *Chlorella vulgaris* obtained in the U.S.’s regulation GRAS status from the Food and Drug Administration (FDA) [325]. Other eucaryotic microalgae such as *Haematococcus pluvidalis*, *Schizochytrium* sp., or *Ulkenia* sp. are recognized as novel food in the EU and obtained GRAS status in the U.S. [326].

Microalgae are employed in a variety of ways for human consumption. They can be incorporated into foodstuffs like dairy products, fish products, cereals, and meat products as whole dry biomass [320,327]. However, in addition to the beneficial effects (antimicrobial activity [328], source of dietary fiber, promotion of growth of probiotics), its application is limited due to the sensory characteristics (namely, fish taste and strong color) [326,329]. The use of microalgal extracts is an effective method for enhancing the nutritional value of food products [330,331]. A third area of potential application of microalgae is the development of functional foods, which may include the incorporation of high-value molecules such as essential amino acids, carbohydrates, pigments, and proteins derived from microalgae [326,332,333].

Microalgae possess a remarkable aptitude for the elimination of a wide spectrum of pollutants and hazardous materials in wastewater, which are produced by various sources, including domestic agricultural runoffs, effluents, textile, printing, pharmaceutical, and electroplating industries [334]. It wasa recently demonstrated that microalgal-mediated wastewater treatment systems have the capacity to reduce antibiotic resistance genes (ARGs) in wastewater [335]. It is hypothesized that microalgae have the capacity to function as natural barriers, thereby playing a pivotal role in hindering the transfer of antibiotic resistance genes (ARGs) between symbiotic bacteria. This hypothesis suggests that microalgae could contribute to a reduction in the abundance of ARGs during the process of wastewater treatment [336]. Conversely, a cyanobacterial bloom caused by the *Planktotrix* and *Microsystis* species at Lake Taihu in China was reported to increase the probability of emergence of antibiotic-resistant bacteria (ARB) [337].

Although a limited number of studies have addressed the issue of antibiotic resistance migration among microalgae and bacteria, Zourou [338] and Nguyen et al. [339] demonstrated that *E. coli* K12 and *E. coli* DH5α are capable of uptake kanamycin resistance genes in co-culture with genetically engineered cyanobacterium *Thermosynechococcus elongatus* BP1. Wang et al.’s [340] findings indicated that cyanobacteria can obtain and transmit ARGs (tetracycline (*tet*A) and sulfonamide (*sul*1) resistant genes) in aquatic environments through HGT. Studies by Li et al. [341] and Inuwa et al. [342] showed that a number of factors, including temperature, pH, the availability of nutrients, UV radiation, and dissolved oxygen, may influence the transfer of ARGs

The aforementioned evidence indicates, despite the beneficial characteristics of microalgae, there is a potential risk of transmission of antibiotic-resistant genes to humans. This is also confirmed by the findings of Cao et al. [343], according to which the transmission of ARGs carried by microalgae to organisms with high nutritional levels within the food web may pose a potential risk to human health. The presence of *Chlorella pyrenoidosa* in their research work was observed to enhance the abundance of ARGs (tetracycline- and sulfonamide-resistant genes), thereby elevating the probability of ARG transmission along the food chain.

### 5.2. Microbiota of Edible Insects and Prevalence of Antibiotic Resistance Genes in Their Bacteria

In Europe and other industrialized countries, entomophagy is often considered to be an unappealing practice. However, in Asia, Africa, Latin America, and Australia, insects are typical components of the human diet. It is estimated that more than 2000 insect species are consumed all over the world [344]. Insects were recently introduced to Europe as novel foods. Commission Implementing Regulations (EU) 2021/1975 [345], (EU) 2022/169 [346], (EU) 2022/188 [347], and (EU) 2023/58 [348] have authorized the placing of frozen, dried, and powdered forms of migratory locust/grasshopper (*Locusta migratoria*), yellow mealworm (*Tenebrio molitor*), house cricket (*Acheta domesticus*), and grain mold beetle, also known as lesser mealworm (*Alphitobius diaperinus*) on the market. Despite the nutritional benefits of consuming insects like high protein and micronutrient content, or the potential antibacterial effects of sterols in edible insect extracts [349], microbiological safety is also a significant factor to be considered, as there is a noticeable lack of research regarding the microbial risks associated with insects for human consumption [350,351].

As outlined by Garofalo et al. [344] and Imathiu [352], the presence of multiple pathogenic bacteria was identified in edible insects. Among the bacterial genera are *Bacillus*, *Campylobacter*, *Clostridium*, *Cronobacter*, *Escherichia*, *Listeria*, *Proteus*, *Pseudomonas*, *Salmonella*, *Serratia*, *Staphylococcus*, *Streptococcus*, *Vibrio*, and *Yersinia*. These genera include emetic, pathogenic, or potentially pathogenic strains characteristic of the microbiota of edible insects. Yeasts and molds are also present in edible insects. As determined by Garofalo et al. [344], *Aspergillus*, *Penicillium*, *Alternaria*, *Chaetomium*, *Mucor*, *Phoma*, *Drechslera*, and *Fusarium* are their typical filamentous fungi. In detail, some xerophilic and potential mycotoxin-producing species were identified, such as *Aspergillus niger* and *Aspergillus flavus/parasiticus*, *Aspergillus ochraceus*, *Penicillium aurantiogriseum*, *Penicillium citrinum*, and *Penicillium verrucosum*. Among the identified yeasts, *Debaryomyces hansenii* is a common species, whereas *Saccharomyces* spp. or *Saccharomyces cerevisiae* are detected in lower frequency. The presence of *Trichosporon asahii*, an opportunistic yeast that causes trichosporonosis in immunocompromised patients, was also confirmed. Gałęcki and Sokół [353] evaluated the presence and the role of edible insects in the transmission of parasitic diseases to humans. They proved that edible insects play an important role in the epidemiology of parasitic diseases in vertebrates. Thus, it was proposed that insect welfare standards and analytical methods should be developed with the objective of minimizing production losses and effectively eliminating pathogens from edible insect farms.

Moreover, recent studies on commercially available edible insects might represent an important reservoir of antibiotic-resistant microorganisms and revealed the presence of some antimicrobial resistance (AR) genes that confer resistance to antibiotics conventionally used in clinical practice [344,354]. A comprehensive review dealing with the role of insects in the acquisition and transmission of antibiotic resistance was prepared by Rawat et al. [355]. Milanović et al. [356] investigated the presence of 11 transferable AR genes in various marketed edible insects and found that among the genes investigated, resistance to tetracycline (*tetK*) occurred with the highest frequency, followed by macrolides (*ermB*) and β-lactamases (*blaZ*). In addition, PCR-based molecular methods have also shown a high prevalence of tetracycline resistance genes in *Hermetia illucens* (the black soldier fly) larvae [357]. In the study of Vandeweyer et al. [358], it was observed that genes conferring resistance to tetracyclines were detected with a high frequency, and insects may carry considerable amounts of AR genes, but the health risk in terms of antibiotic resistances is comparable to other food matrices. Nevertheless, Osimani et al. [359] also discovered that the presence of various *tet* genes in organic wheatmeal, larvae, and frass significantly had contributed to the transmission of AR genes and/or antibiotic-resistant microorganisms in larvae, even in the absence of selective pressure exerted by antibiotics. Among others, tetracycline was categorized by the WHO [360] as a critically important antibiotic for clinical usage; thus, the presence of this antibiotic could increase the possibility of ARG spread among bacteria.

A summary of the data indicates that the microorganisms identified in the microbiota of edible insects may present a risk to consumers. Moreover, the presence of antibiotic resistance genes could potentially exacerbate the spread of antibiotic resistance, which is already a significant concern.

## 6. Conclusions

The food consumed by humans almost always contains a number of microorganisms, the presence of which affects the quality and safety of our food. In many instances, the introduction of pathogens into the human body via foodstuff results in the emergence of health complications, attributable to the pathogenicity factors inherent to these microorganisms. However, the treatment of bacterial infections also presents a significant challenge due to the antibiotic resistance encoded within the bacterial genome. Furthermore, it was demonstrated that resistance to antibiotics can be transferred to non-pathogenic microorganisms, as evidenced by a substantial amount of literature from recent years.

Nevertheless, the direction of transfer of resistance is not one-way. Experimental evidence indicates that lactic acid bacteria, which are instrumental in fermentation processes, can also possess resistance genes and transfer them to pathogenic bacteria. This phenomenon enhances the resistance of the pathogenic bacteria to antibiotics, thereby complicating the fight against them.

Despite the emphasis placed on the role of animal foods in the scientific literature regarding the spread of antibiotic resistance, this review demonstrates that foods of plant origin play as important of a role in the spread of antibiotic resistance as those of animal origin. As demonstrated in this review article, a range of food items, also including drinking water, and even insects and microalgae belonging to the category of novel foods, carry microbes that require increased attention due to the presence of resistance genes in their genomes. It is also crucial to consider that the expression of genes can be enhanced or repressed under specific conditions. Therefore, it is essential to prioritize the production and storage of food under conditions that not only do the reproduction of harmful microbes inhibit but also suppress the expression of their resistance genes.

In the course of preparing this review article, it became evident that a considerable proportion of the extant publications in the literature fail to provide adequate clarification with regard to the nature of the resistance in question. In many cases, the authors refer to antimicrobial resistance, even in instances where their research has focused on bacteria and antibiotics. To address this issue, it is recommended that authors place greater emphasis on the precise designation of the topic, thereby facilitating a more efficient search and reference to the results of their work by other researchers. Furthermore, challenges were encountered during the collection of data pertaining to the presence of specific microorganisms in various food types, the antibiotics to which they are resistant, and the genes responsible for this resistance. Frequently, only partial results are disseminated in published articles, thus necessitating a more comprehensive, multifaceted analysis of the subject area in question, accompanied by a more extensive presentation of the data.

As demonstrated in our review article, the spread of antibiotic resistance among pathogenic and non-pathogenic bacteria is becoming increasingly significant, representing a mounting challenge in the food industry.

In order to address this challenge, it is imperative to implement strategies aimed at mitigating the prevalence of antibiotic-resistant bacteria, while concomitantly diminishing the environmental conditions conducive to their proliferation (e.g., by reducing antibiotic presence in the environment, thereby attenuating selective pressure). This can be achieved through the implementation of various solutions, including the utilization of bacteriophages or parasitic bacteria within the food industry, along with the incorporation of antibacterial compounds derived from natural sources. Despite the fact that this area of research is growing rapidly, it continues to offer novel insights and remains a worthwhile focus for further investigation.

As the use of antibiotics as growth promoters and prophylactic agents is a typical manifestation of inappropriate antibiotic use in food producing animals, the development of an effective veterinary antibiotic policy can significantly contribute to reducing the use of antibiotics. It is imperative that antibiotics are applied on farms only when recommended by a veterinary professional and under their direct supervision. Furthermore, veterinarians must play a pivotal role in raising awareness among farmers regarding the significance of appropriate antibiotic usage through effective information and education. In food animal production, alternatives to antibiotics are of significant importance in the reduction in antibiotic usage. Such alternatives include the utilization of functional feed additives, probiotic bacteria, among others. Nevertheless, it is imperative to note that in food processing, the employment of probiotic or technological microorganisms that lack the capability of horizontal gene transfer is inevitable.

In order to control the emergence and spread of antibiotic resistance in the food chain, it is important that the improvement of their use in primary food production is prioritized, in conjunction with ensuring a safe and high-quality food supply.

By fostering collaboration across disciplines and implementing effective monitoring and treatment strategies, we can significantly reduce the prevalence of antibiotic-resistant bacteria and ensure safer food for all. Collective action and vigilance are essential in this fight against antibiotic resistance, paving the way for a healthier future.

## Figures and Tables

**Table 3:** Categories of novel food according to Regulation (EU) 2015/2283 [306] and examples [311,312,313,314].

Categories of Novel Food	Examples
Foods with new or modified molecular structure	D-Tagatose, salatrim
Foods consisting of, isolated from or produced from material of mineral origin	clinoptilolite (zeolite)
Foods consisting of, isolated from or produced from microorganisms, fungi, algae	algae oil from the microalgae *Ulkenia* sp.
Foods consisting of, isolated from or produced from plants or their parts	noni juice (*Morinda citrifolia*), chia seeds (*Salvia hispanica*)
Foods consisting of, isolated from or produced from animals or their parts	insects, oil from Antarctic krill (*Euphasia superba*), peptides from the fish *Sardinops sagax*
Food consisting of, isolated from or produced from cell culture or tissue culture derived from animals, plants, micro-organisms, fungi, or algae	extract from cell cultures of *Echinacea angustifolia*, in vitro meat
Food resulting from a production process not used for food production within the Union before 15 May 1997	high pressure pasteurized fruit preparations, UV-treated food: mushrooms (*Agaricus bisporus*), baker’s yeast (*Saccharomyces cerevisiae*), bread, milk
Food consisting of engineered nanomaterials	nanosilver provides antimicrobial properties to food packaging, nanocapsules (containing flavor or color enhancers, or added vitamins)
Vitamins, minerals and other substances used in accordance with Directive 2002/46/EC, Regulation (EC) No 1925/2006 or Regulation (EU) No 609/2013	iron (II) ammonium phosphate, vitamin K2 (menaquinone), chromium picolinate
Food used exclusively in food supplements within the Union before 15 May 1997	maqui berry (Aristotelia chilensis), rose root (Rhodiola rosea)

## Data Availability

No new data were created or analyzed in this study.
